# Affective and cognitive behavior is not altered by chronic constriction injury in B7-H1 deficient and wildtype mice

**DOI:** 10.1186/s12868-019-0498-4

**Published:** 2019-04-11

**Authors:** Franziska Karl, Maria B. Nandini Colaço, Annemarie Schulte, Claudia Sommer, Nurcan Üçeyler

**Affiliations:** Department of Neurology, University of Würzburg, Josef-Schneider-Str. 11, 97080 Würzburg, Germany

**Keywords:** B7-H1, Immune system, CCI, Anxiety, Cognitive behavior

## Abstract

**Background:**

Chronic neuropathic pain is often associated with anxiety, depressive symptoms, and cognitive impairment with relevant impact on patients` health related quality of life. To investigate the influence of a pro-inflammatory phenotype on affective and cognitive behavior under neuropathic pain conditions, we assessed mice deficient of the B7 homolog 1 (B7-H1), a major inhibitor of inflammatory response.

**Results:**

Adult B7-H1 ko mice and wildtype littermates (WT) received a chronic constriction injury (CCI) of the sciatic nerve, and we assessed mechanical and thermal sensitivity at selected time points. Both genotypes developed mechanical (*p* < 0.001) and heat hypersensitivity (*p* < 0.01) 7, 14, and 20 days after surgery. We performed three tests for anxiety-like behavior: the light–dark box, the elevated plus maze, and the open field. As supported by the results of these tests for anxiety-like behavior, no relevant differences were found between genotypes after CCI. Depression-like behavior was assessed using the forced swim test. Also, CCI had no effect on depression like behavior. For cognitive behavior, we applied the Morris water maze for spatial learning and memory and the novel object recognition test for object recognition, long-, and short-term memory. Learning and memory did not differ in B7-H1 ko and WT mice after CCI.

**Conclusions:**

Our study reveals that the impact of B7-H1 on affective-, depression-like- and learning-behavior, and memory performance might play a subordinate role in mice after nerve lesion.

## Background

Chronic neuropathic pain, caused by lesions or diseases of the somatosensory nervous system [1], is often associated with anxiety and depressive symptoms [2] and considerably impacts on patients` health related quality of life [3]. Neuro-immune interactions contribute to the development and maintenance of neuropathic pain [4]. An imbalance between pro- and anti-inflammatory systems seems pathophysiologically relevant [5]. Pro-inflammatory cytokines and other inflammatory mediators are also modifiers of mood and cognition, leading to depressive symptoms and memory deficits in patients and respective behavior in animal models [6, 7].

One relevant candidate among immune mediators is B7 homolog 1 (B7-H1; synonyms: PD-L1, CD274), a type 1 transmembrane protein and a member of the B7/CD28 family [8, 9]. B7-H1 is expressed on non-lymphoid tissue, on activated macrophages, and on dendritic cells and is one of the two ligands of the programmed-death receptor-1 (PD-1) [9]. The interaction of B7-H1 with its receptor inhibits T cell proliferation and cytokine production [10]. Using the chronic constriction injury (CCI), we showed that B7-H1 deficiency leads to an excessive pro-inflammatory response and sustained and enhanced pain behavior after nerve injury [11], whereas no differences were observed between genotypes in pain behavior after spared nerve injury (SNI) [12]. Additionally, a recent study identified B7-H1 as an endogenous pain inhibitor in a neuropathic and bone cancer pain model [13].

Here we set out to investigate affective behavior, learning performance, and memory of B7-H1 ko and wildtype (WT) littermates under neuropathic pain conditions. Using CCI we hypothesized that B7-H1 ko mice develop more anxiety-like behavior and cognitive deficits after nerve lesion compared to WT mice based on their pro-inflammatory phenotype.

## Methods

### Ethics statement

All experiments were approved by the Ethics Committee for Animal Research of the Bavarian State authorities (Regierung von Unterfranken, #3/12). Mice were housed in the animal facilities of the University of Würzburg (Department of Neurology; Center for Experimental Molecular Medicine) with food and water access ad libitum. Animal use and care were in accordance with the institutional guidelines.

### Animals

We investigated 119 male mice at the age of 8–12 weeks. B7-H1 ko mice were generated by L. Chen, Baltimore, USA [14] and inbred WT littermates of C57Bl/6J background served as controls. Mice were housed in a reversed light–dark cycle (light cycle: 7 pm–7 am; dark cycle: 7 am–7 pm).

### Behavioral testing

**Fig. 1:**
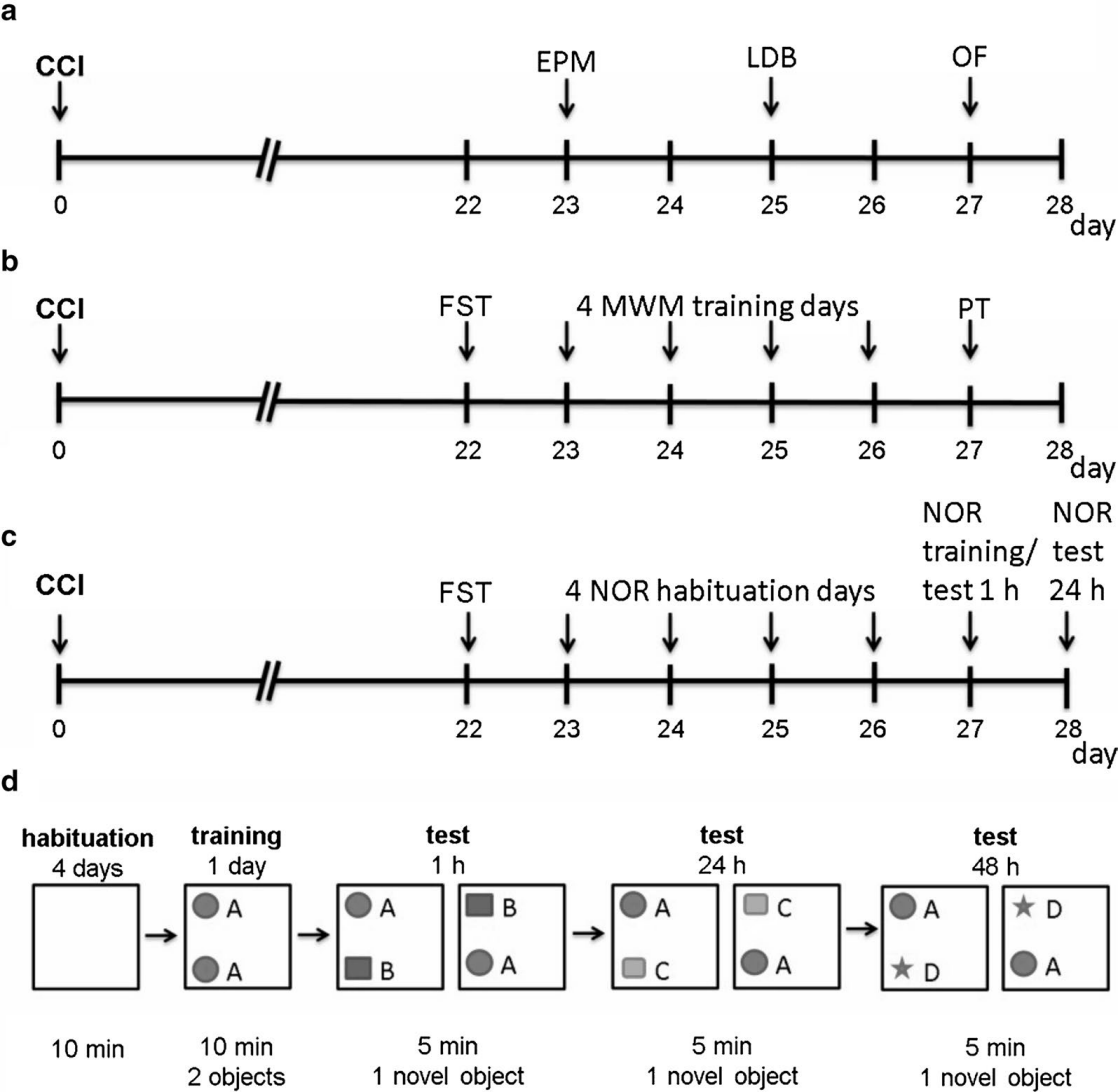
Experimental design of tests for affective and cognitive behavior after chronic constriction injury (CCI). **a** Mice were tested in three paradigms for anxiety-like behavior in the following sequence: elevated plus maze (EPM), light–dark box (LDB), and open field (OF). **b** Mice were tested in the forced-swim test (FST) for depression-like behavior and in the Morris water maze test (MWM) and Morris water maze probe trial (PT) for learning and memory. **c** Mice performed the FST and the novel object recognition test (NOR) for cognition. **d** Detailed test procedure of the NOR. After habituation on four consecutive days (10 min each) in the open field arena, mice were familiarized with two similar objects in the training phase of 10 min. In the test phase, mice faced one familiar and one novel object after 1 h (short-term memory), and 24 h (long-term memory) for 5 min, each

An experienced investigator blinded to the genotype performed all behavioral tests. Mice were assessed during their active phase under red light. Tests were performed in a black box to avoid interference with other mice and the investigator. The experimental algorithms are shown in Fig. 1a–c. All tests were video recorded for off-line analysis (see below). Behavioral tests were performed as described previously [15].

#### Mechanical and thermal sensitivity

For obtaining baseline values and allowing the animals to adapt to the testing apparatus, all animals were investigated twice before surgery. After chronic constriction injury (CCI, see below), behavioral tests were performed at defined time points (7, 14, and 21 days, n ≥ 7 mice/genotype). The paw withdrawal thresholds upon mechanical stimulation were investigated using the up-and-down-method for the von-Frey test [16]. Mice were placed in plexi glass cages on a wire mesh. After 45 min adaption, the plantar surface of the hind paws was touched with a von-Frey filament starting at 0.69 g. If the mouse reacted with hind paw withdrawal, the next finer von-Frey filament was used. If the mouse did not react, the next thicker von-Frey filament was used. Each hind paw was tested six times. The 50% withdrawal threshold (i.e. force of the von Frey hair to which an animal reacts in 50% of the administrations) was calculated.

The Hargreaves method applying a standard Ugo Basile Algesiometer (Comerio, Italy) was used to determine the sensitivity to heat stimuli [17]. Mice were placed on a glass surface surrounded by a plexi glass box. After 45 min adaption, a radiant heat stimulus (25 IR) was applied to the plantar surface of the hind paw and the withdrawal latency was automatically recorded. A stimulus cut-off time of 16 s was observed to prevent tissue damage by heat. Each hind paw was consecutively tested three times.

#### Anxiety- and depression-like behavior

To assess the intra-individual variation in affective behavior, three different tests were applied for anxiety-like behavior: light–dark box (LDB) [18], elevated plus maze (EPM) [19], and open field (OF) [20]. EPM and OF were additionally used to investigate exploratory behavior of the mice. Each mouse was tested once for 5 min in each apparatus.

The LDB consisted of an illuminated (40 cm × 20.5 cm) and a dark compartment (40 cm × 19.5 cm). As a starting point, mice were placed in the light box. Mice could freely explore the apparatus and choose between the two inter-connected compartments. The entries into the light and dark box, and the percentage of time spent in the light and dark box were recorded.

The EPM apparatus consisted of two opposite open arms (66.5 cm) and two closed arms (65.5 cm), separated by a junction area. Mice were individually placed in the middle of the apparatus, facing an open arm. The entries into open and closed arms, and the total time spent in open and closed arms were determined.

The OF (40 cm × 40 cm) consisted of two areas: the center zone (20 cm × 20 cm) and the surrounding area. Mice were placed individually in the middle of the center zone; entries into the center zone and the distance and the time travelled in the center zone were recorded. For exploratory behavior, we additionally determined the total distance travelled in the open field apparatus.

For depression-like behavior we performed the forced-swim test (FST) [21]. Mice were placed in a glass cylinder filled with water (diameter of cylinder: 11.5 cm; water height: 12.5 cm; water temperature: 20 °C ± 2 °C). Within a six min testing phase, time spent immobile during five min of observation was determined.

#### Cognitive behavior

For the investigation of learning behavior and memory we performed the Morris water maze test (MWM) [22]. Mice were investigated in a cylindrical plastic pool (diameter: 118.5 cm), filled with opaque water (temperature: 20 °C ± 2 °C) just covering the platform (diameter: 8 cm). The pool was divided into four quadrants and the platform was placed in the middle of one quadrant (target quadrant). Mice had six trails daily with different starting points on four consecutive training days. The time mice needed to reach the platform was measured and daily average time for every group was calculated. If the mice were not able to find the platform within 60 s they were placed on the platform for 15 s for orientation in the pool. Additionally, we determined the total distance travelled and the average speed. For memory performance, the MWM probe trial (PT) was performed on the fifth day, during which the platform was removed. A new starting point on the opposite side of the target quadrant was chosen. The total distance travelled, the average speed, the distance travelled in the target quadrant, and the time mice spent in the target quadrant were measured during a 30 s observation period.

As a further test for cognition, we performed the novel object recognition (NOR) test, which considers the differences in the exploration time of novel and familiar objects [23]. For habituation to the apparatus, mice were placed into the open field arena without objects for 10 min on four consecutive days. On the following training day, mice were familiarized with two identical objects in the open field arena for 10 min. In the test phase one object was identical to the training object and one was novel. During the training and the test phase objects were located in opposite and symmetrical corners. The location of the familiar and the novel object was counterbalanced. Mice underwent two different test phases (Fig. 1d); 1 h after training as a test for short-term memory, and 24 h after training for long-term memory [24]. To determine the locomotor activity, we measured the total distance, and the average speed mice travelled during the training and the two tests. We investigated the time mice explored familiar (T_F_) and novel objects (T_N_) during the test phases, and calculated the recognition index: T_N_/(T_N_ + T_F_) [25]. Exploration of an object was defined as orientation of the animals nose at a distance of < 2 cm to the object. Sitting on the object was not recorded as exploration.

### Chronic constriction injury (CCI)

Naïve mice were anesthetized with intraperitoneal barbiturate (Narcoren^®^, 50 mg/kg body weight) injections. CCI or a sham surgery was performed as described earlier [26] with slight modifications [27]. Skin was incised and the sciatic nerve was exposed by a blunt dissection through the biceps femoris muscle. Three loosely tied ligatures (7-0 prolene), with a distance of 1 mm between each ligature were placed around the sciatic nerve proximal to the trifurcation. Incisions were closed with muscle and skin sutures. In sham surgery, the sciatic nerve was exposed, but not ligated. After surgery, mice were kept at 37 °C until they regained consciousness. CCI, inducing thermal hyperalgesia and mechanical allodynia, was used as an established method for neuropathic pain [26]. Behavioral experiments were conducted 22–29 days after surgery. At the end of the experiments, mice were euthanized by decapitation in deep isoflurane anesthesia (5% isoflurane via a vaporizer).

### Video processing and statistical analysis

Recorded videos were analyzed using the ANY-maze video tracking software (system version: 4.99 m, Stoelting, USA). For statistical analysis and graph design SPSS IBM software version 24 was employed (Ehningen, Germany). We applied the non-parametric Mann–Whitney U test, since data were not normally distributed in the Kolmogorov–Smirnov test. The Bonferroni-Holm procedure was applied to correct for multiple comparisons as appropriate. Data are illustrated as bar graphs (GraphPad Prism, software version 5.03, San Diego, CA, USA), displaying the mean with standard error of the mean (SEM). Data were stratified for treatment groups (naïve, sham, and CCI). P values < 0.05 were considered statistically significant.

## Results

### Mechanical and heat hypersensitivity in B7-H1 ko and WT mice after CCI

**Fig. 2:**
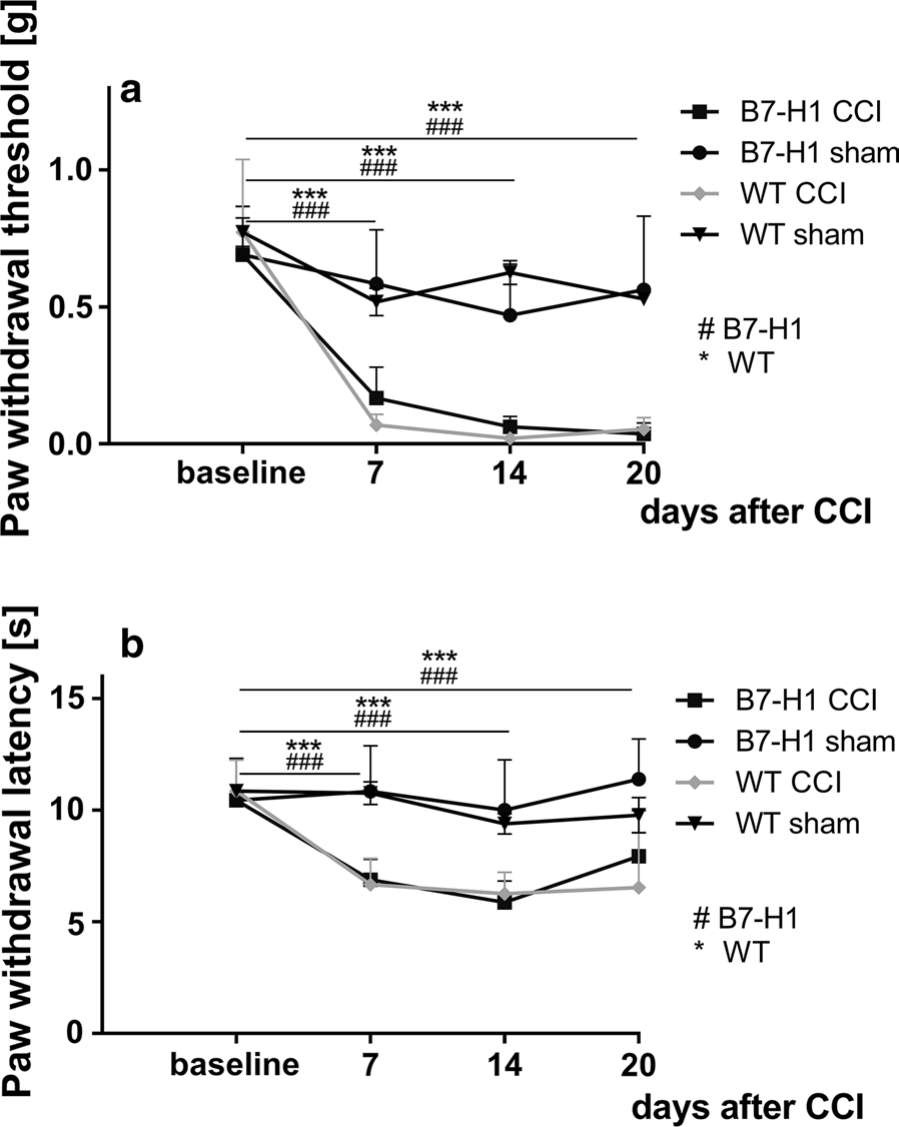
Mechanical and thermal sensitivity after chronic constriction injury. B7-H1 ko and WT mice developed hypersensitivity to mechanical stimuli 7, 14, and 20 days after CCI (*p* < 0.001). Both genotypes developed heat hypersensitivity 2, 14, and 20 days after CCI. B7-H1 ko: CCI: 15 male mice; sham: 13 male mice. WT: CCI: 14 male mice; sham: 12 male mice. ***p < 0.001. ^###^*p* < 0.001

Mechanical withdrawal thresholds did not differ at baseline. After CCI, B7-H1 ko and WT mice developed mechanical hypersensitivity 7, 14, and 20 days without differences between genotypes (*p* < 0.001 each, Fig. 2a). No difference was detected in heat withdrawal latencies between genotypes at baseline, and both genotypes developed heat hypersensitivity after CCI (*p* < 0.01, Fig. 2b).

### No influence of CCI on anxiety- and depression-like behavior

**Fig. 3:**
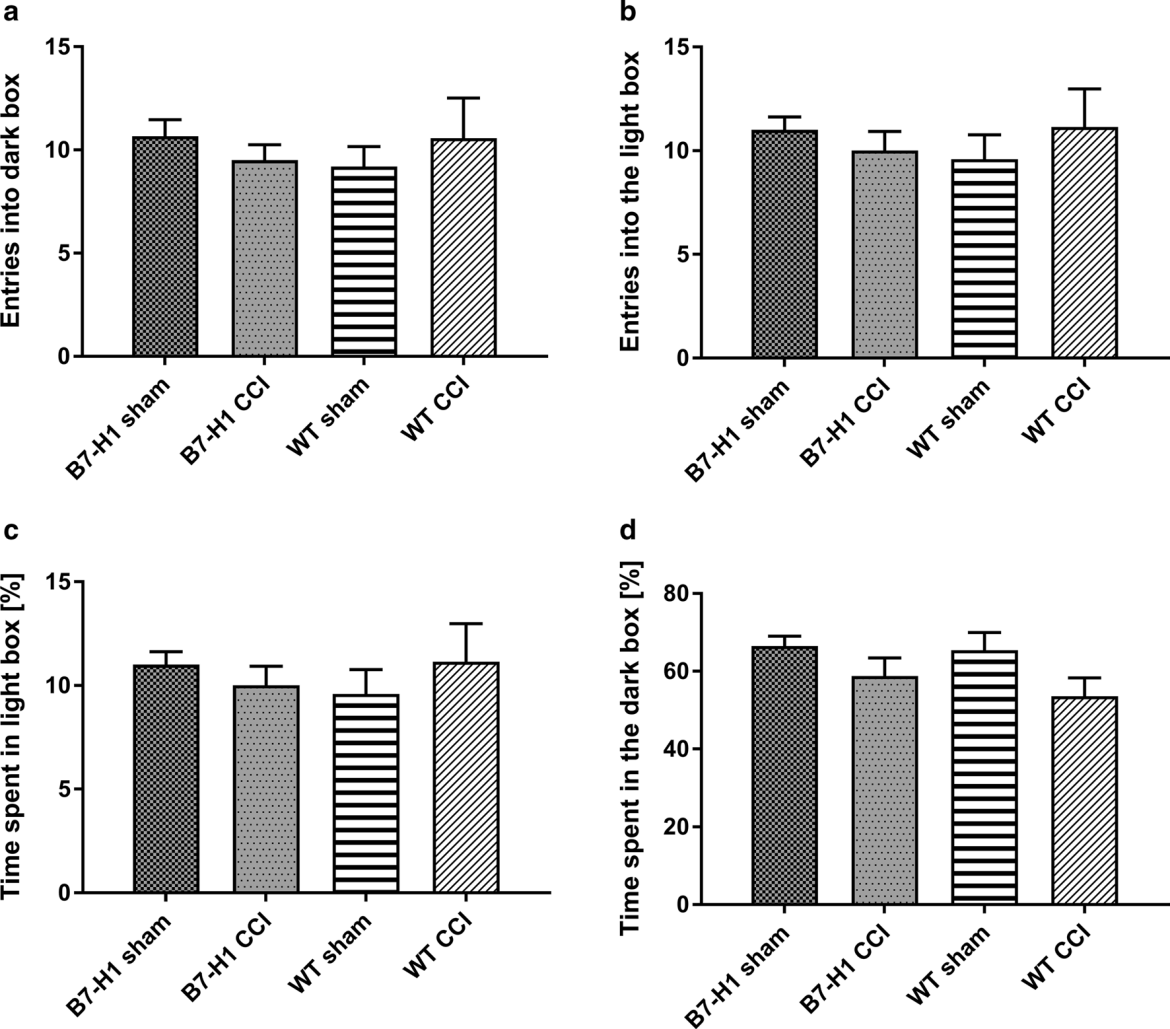
Anxiety-like behavior in the light-dark box (LDB). Bar graphs show the results of the LDB in B7-H1 ko and wildtype littermates (WT). Mice were investigated after chronic constriction injury (CCI) and sham surgery. No differences were found after CCI in the number of entries into the dark box (**a**) and into the light box (**b**). Also, time spent in the light box (**c**) and the time mice spent in the dark box (**d**) did not differ between genotypes and surgeries. B7-H1 ko: sham: 6 males, CCI: 8 males. WT: sham: 6 males, CCI: 7 males

In the LDB, B7-H1 ko and WT mice did not differ in the number of entries into the dark box, entries into the light box, and in the time mice spent in the light or dark box (Fig. 3).

**Fig. 4:**
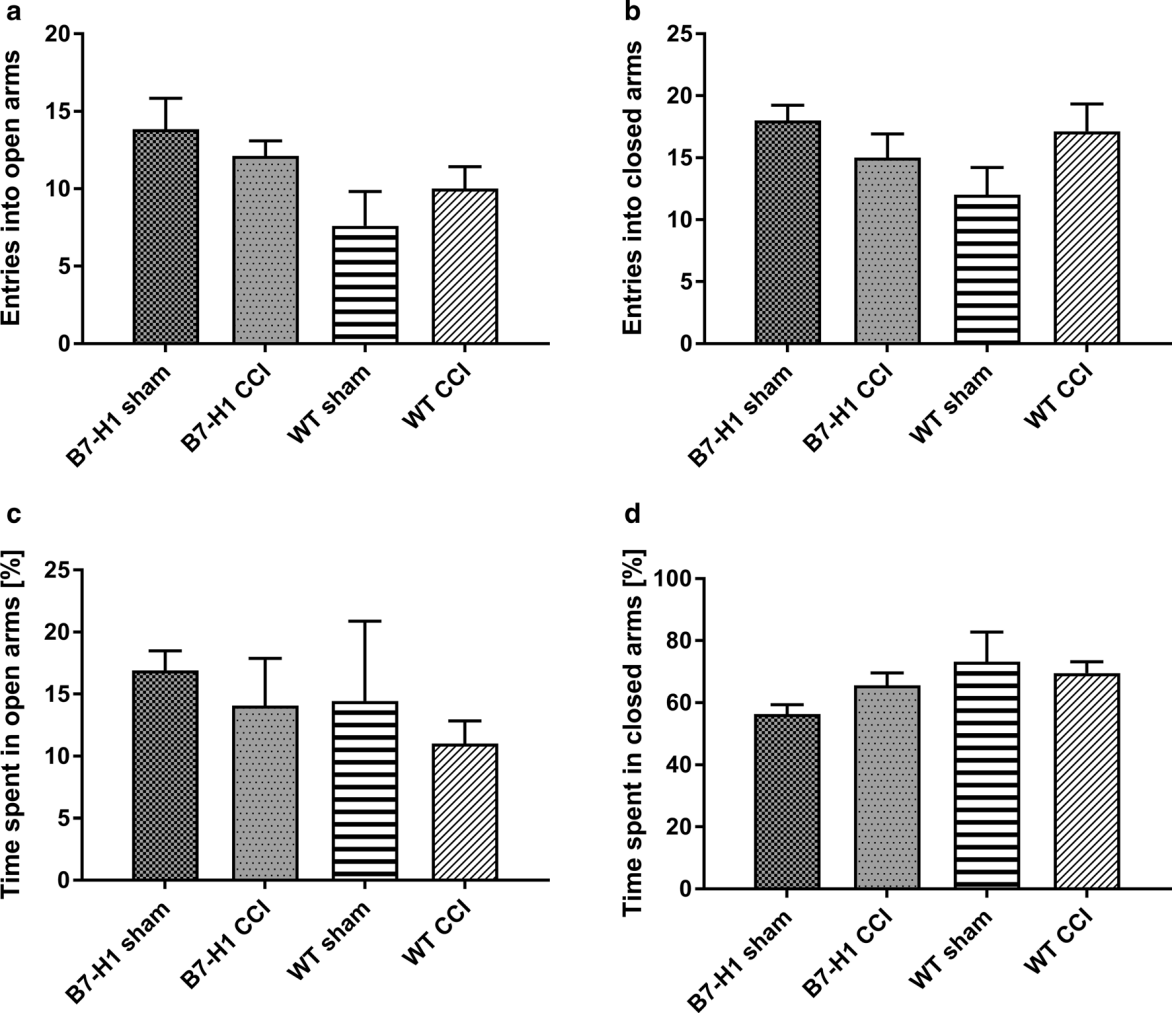
Anxiety-like behavior in the elevated plus maze (EPM). Bar graphs show the results of the EPM in B7-H1 ko and wildtype littermates (WT). Mice were investigated after chronic constriction injury (CCI) and sham surgery. CCI did not lead to differences in entries into the open arms (**a**) and entries into the closed arms (**b**) in B7-H1 ko and WT mice. Both genotypes did not differ in time spent in open arms (**c**) and time spent in closed arms (**d**) after CCI. B7-H1 ko: sham: 6 males, CCI: 8 males. WT: sham: 6 males, CCI: 6 males

In the EPM, entries into the open arms and entries in the closed arms did not differ between genotypes and surgeries (Fig. 4b). No differences were found for both genotypes in time spent in the closed and open arms (Fig. 4c, d).

**Fig. 5:**
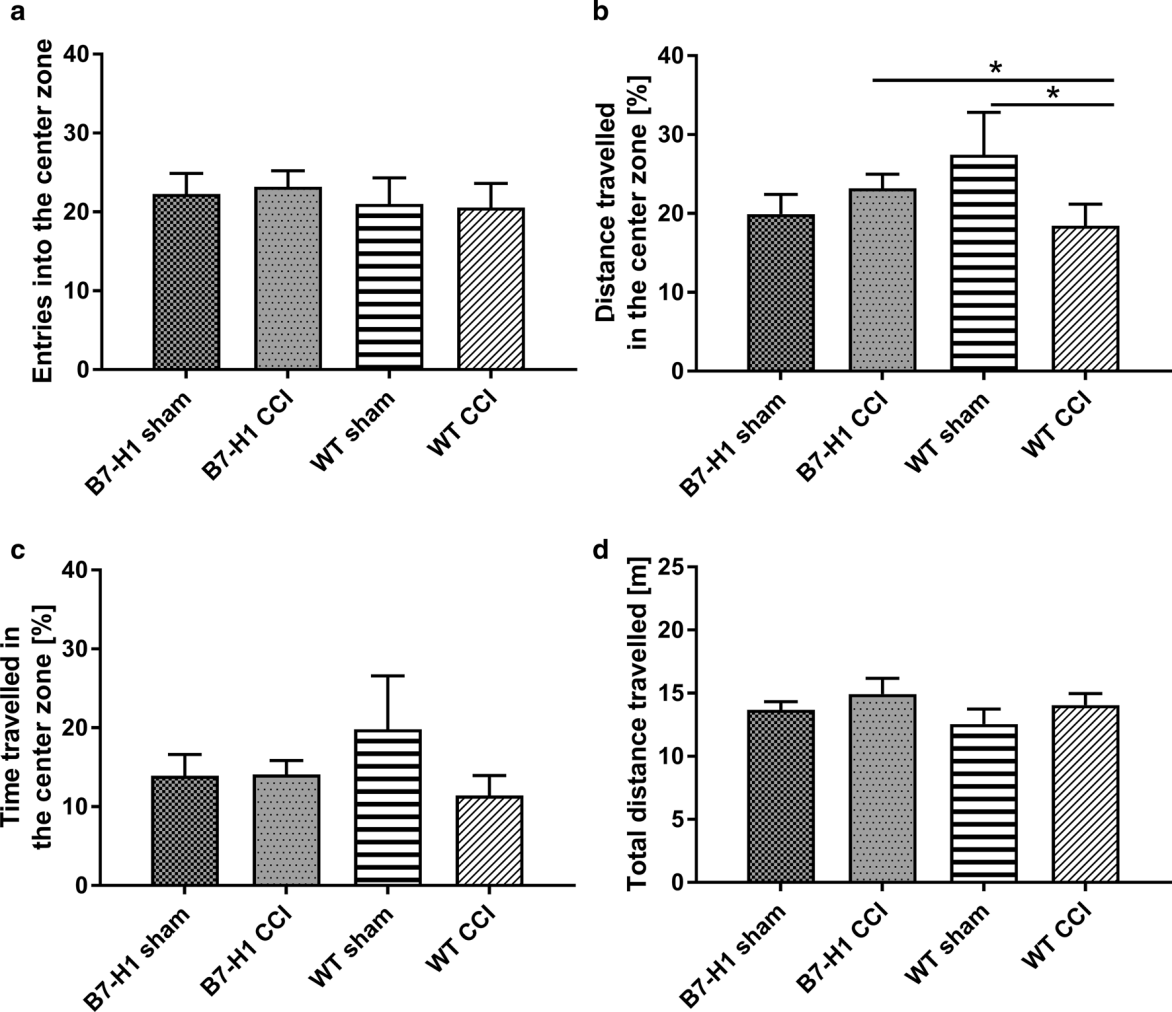
Anxiety-like behavior and locomotor activity in the open field (OF). Bar graphs show the results of the OF test. B7-H1 ko and wildtype littermates (WT) were investigated naïve, after chronic constriction injury (CCI), and sham surgery. No difference was found between genotypes and surgeries in the number of entries into the center zone (**a**). **b** B7-H1 ko mice travelled more after CCI compared to WT mice (*p* < 0.05). WT mice travelled less after CCI compared to sham surgery (*p* < 0.05). **c** No difference in time travelled in the center zone was detected. **d** Total distance travelled did not differ between genotypes and surgeries. B7-H1 ko: sham: 14 males, CCI: 22 males. WT: sham: 11 males, CCI: 20 males. *p < 0.05

In the OF, as a further test for anxiety-like behavior, time spent in the center zone did not differ between genotypes or surgery groups (Fig. 5a). After CCI, B7-H1 ko travelled longer distances in the center zone compared to WT mice (*p* < 0.05 each, Fig. 5b). Only WT mice were affected by CCI and travelled less in the center zone after surgery compared to WT mice after sham surgery (*p* < 0.05, Fig. 5b). No differences were found between genotypes after CCI. The exploratory behavior, as measured by the total distance travelled in the open field, did not differ between genotypes and surgeries (Fig. 5d).

**Fig. 6:**
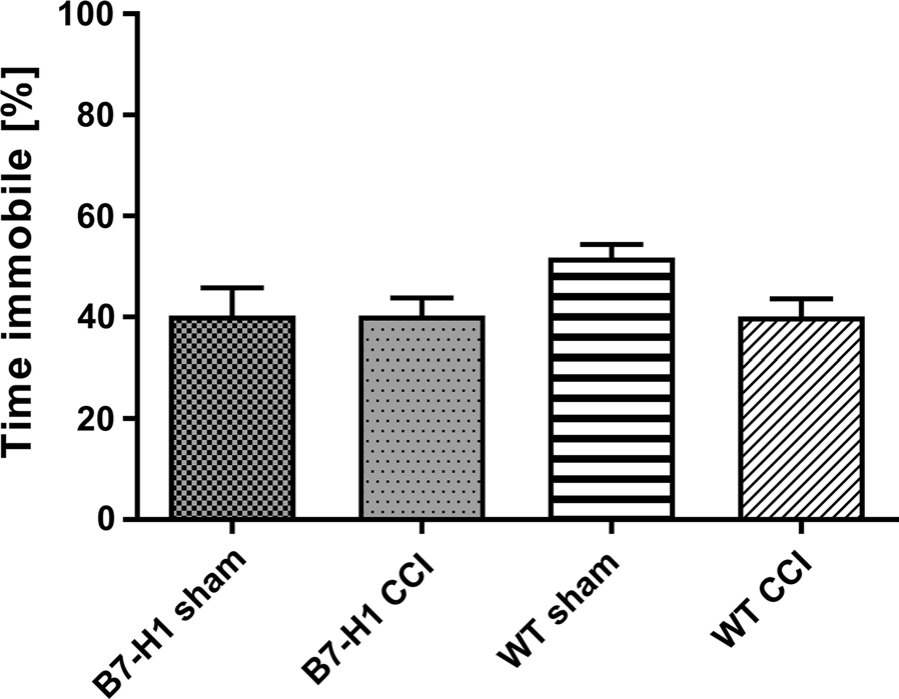
Depression-like behavior in the forced-swim test. Bar graphs show the results of B7-H1 ko and wildtype littermates (WT), after chronic constriction injury (CCI) and sham surgery. Time spent immobile did not differ between genotypes and surgeries. B7-H1 ko: sham: 14 males, CCI: 20 males. WT: sham: 10 males, CCI: 18 males

In the FST, B7-H1 ko and WT mice did not show any hints for depression-like behavior after CCI (Fig. 6).

### No influence of CCI on learning behavior and memory

**Fig. 7:**
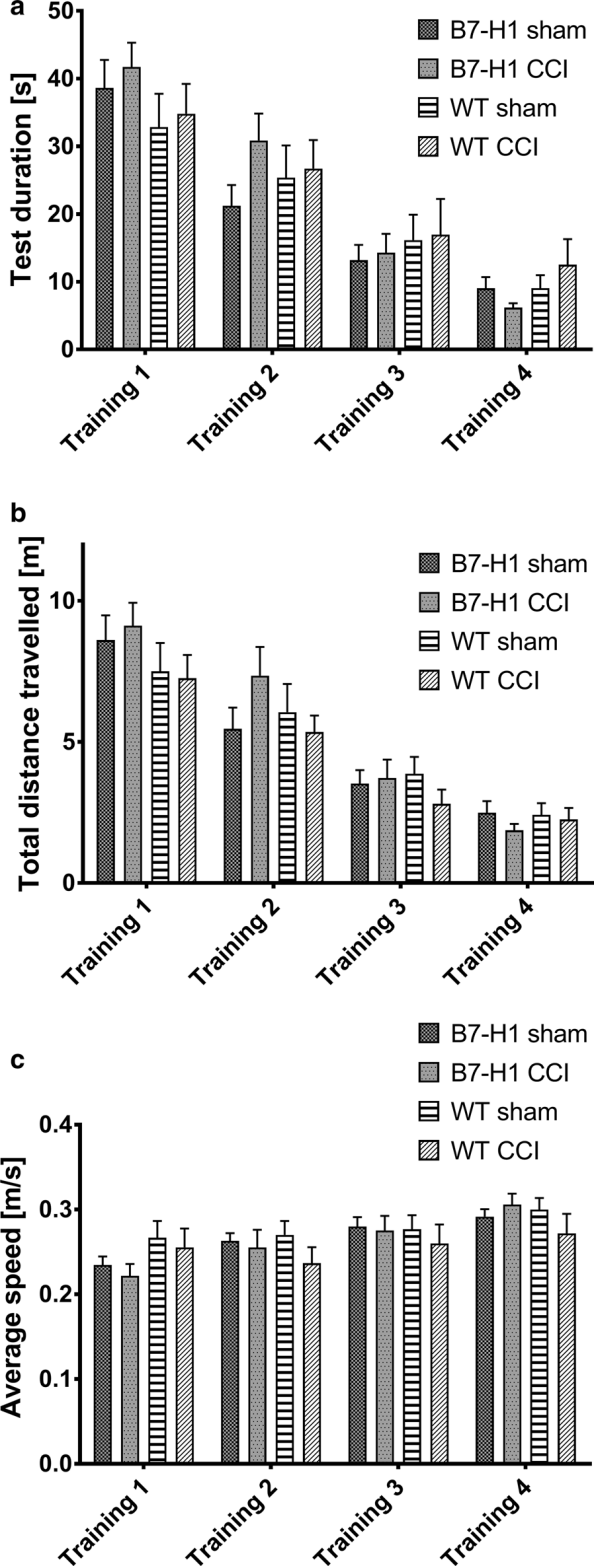
Cognitive behavior in the Morris water maze (MWM). Bar graphs show the results of the training in the MWM of B7-H1 ko and wildtype littermates (WT), after chronic constriction injury (CCI) of the right sciatic nerve and after sham surgery. Mice were tested on four consecutive days. Time mice needed to reach the platform did not differ between genotypes and surgeries (**a**). No differences were found in the total distance travelled between genotypes and treatment (**b**). The average speed did not differ between genotypes and surgeries (**c**). B7-H1 ko: sham: 14 males, CCI: 14 males. WT: sham: 14 males, CCI: 14 males

Time mice spent to reach the hidden platform in the MWM was similar in both genotypes and did not change after surgery. Also, the slope of the learning curves (i.e. decrease in test duration over time) did not differ between genotypes and sham or CCI surgery (Fig. 7a). No relevant differences were found in the total distance and in the average speed mice swam in the MWM during the training days (Fig. 7b, c).

**Fig. 8:**
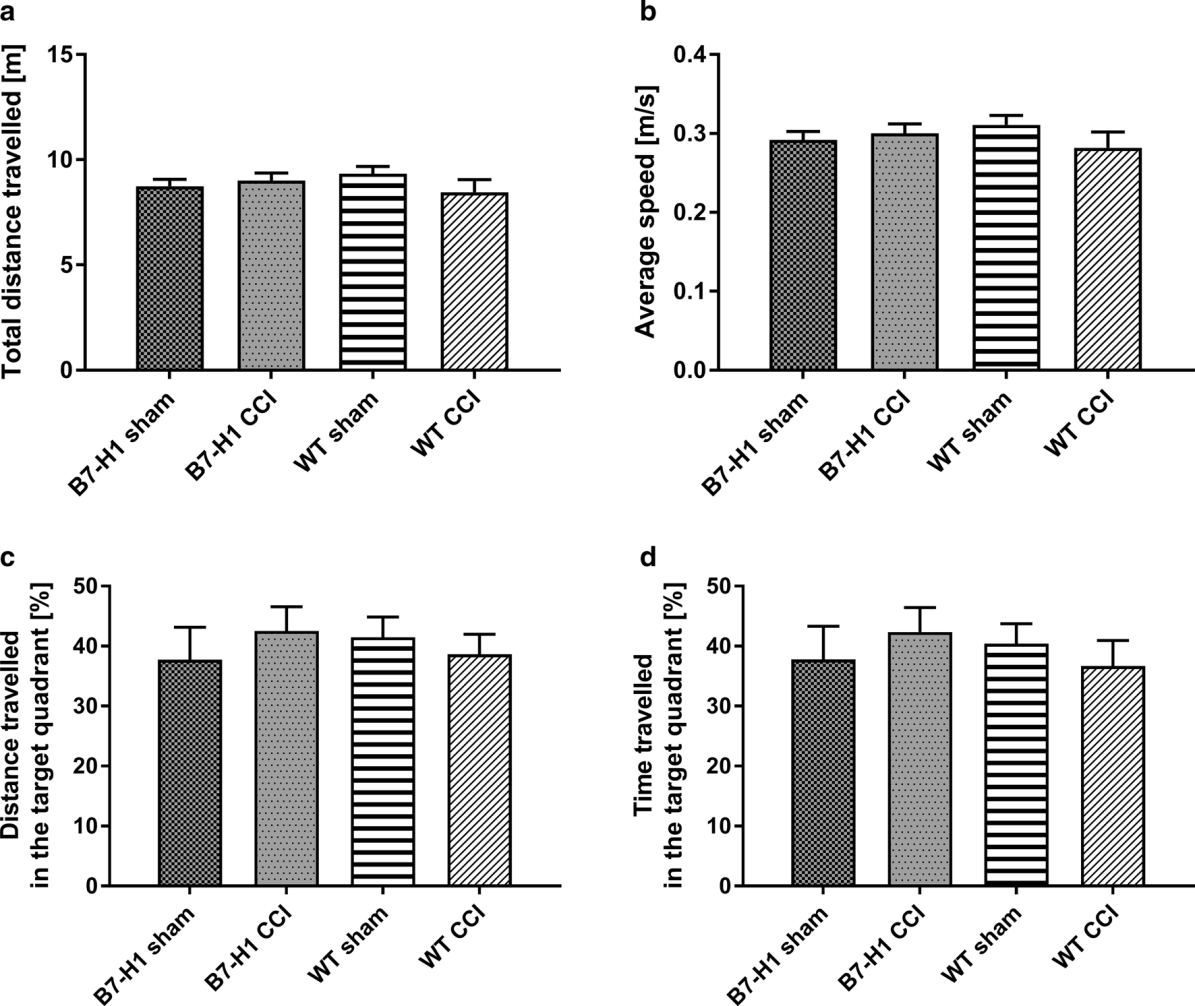
Memory and locomotor impairment in the Morris water maze (MWM). Bar graphs show the results of the in the MWM. B7-H1 ko and wildtype littermates (WT) were investigated naïve, after chronic constriction injury (CCI) of the right sciatic nerve and after sham surgery. No differences were found between genotypes and surgeries in the total distance travelled (**a**), the average speed (**b**), the distance travelled in the target quadrant (**c**), and the time travelled in the target quadrant (**d**). B7-H1 ko: sham: 14 males, CCI: 14 males. WT: sham: 14 males, CCI: 14 males

The total distance travelled and the average speed did not differ between genotypes and surgeries (Fig. 8a, b). No differences were found in the distance travelled, and in the time mice spent in the target quadrant between B7-H1 and WT mice after sham, or CCI surgery (Fig. 8c, d).

### B7-H1 mice show higher preference to novel objects after CCI

**Fig. 9:**
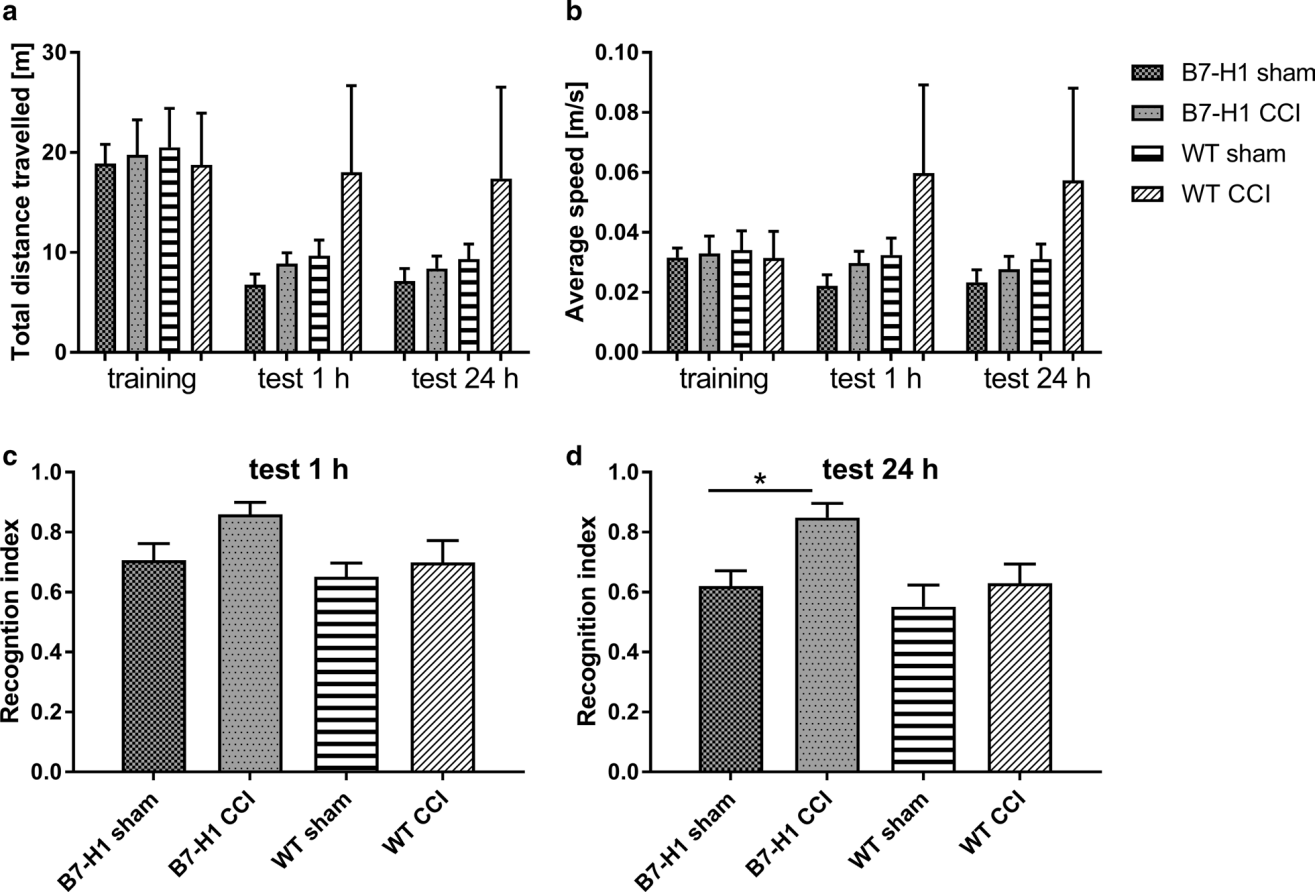
Locomotor activity, short-term, and long-term memory in the novel object recognition test (NOR). Bar graphs show the results of the NOR of B7-H1 ko and WT mice after CCI, and sham surgery. No differences were found between genotypes and surgery groups in the total distance travelled (**a**), and the average speed (**b**). **c** The recognition index did not differ between B7-H1 ko and WT mice, and between sham, and CCI surgery 1 h after training. **d** B7-H1 ko mice did not show differences in long-term memory (24 h after trainng) after surgery, whereas WT mice displayed higher preference towards the novel object after CCI compared to WT mice after sham surgery (*p* < 0.05). B7-H1 ko: sham: 8 males, CCI: 8 males. WT: sham: 6 males, CCI: 6 males. *p < 0.05

Both genotypes showed similar locomotor activity, as measured by the total distance travelled and the average speed during the training and the test phases (Fig. 9a, b). Even in the short-term memory test 1 h after the training, CCI and sham operated B7-H1 ko and WT mice did not differ (Fig. 9c). However, in the test for long-term memory (24 h after the training) B7-H1 ko mice displayed a higher recognition index compared to sham operated B7-H1 ko mice. WT mice did not show intergroup differences after CCI surgery compared to sham operated mice (Fig. 9d).

## Discussion

We characterized the affective-, depression-like- and learning-behavior, and memory performance of B7-H1 ko mice after CCI to investigate if the pro-inflammatory phenotype of B7-H1 ko mice leads to altered anxiety-like behavior and cognitive impairment under neuropathic pain conditions. Our data give no hints for relevant differences between B7-H1 ko and WT mice, since both genotypes did not develop anxiety-, depression-like behavior or cognitive impairment after CCI. Thus, the pathophysiological influence of a pro-inflammatory phenotype on affective and cognitive behavior after nerve lesion seems subordinate in B7-H1 mice.

Patients with chronic pain frequently report of anxiety, depression, or cognitive deficits [28, 29]. Similarly, hints for cognitive impairment were found in animal models of chronic pain [30, 31]. In several studies investigating rats, mostly an increase in anxiety and depression-like behavior was reported after nerve injury [32, 33]. In contrast, data obtained in mice are conflicting such as partial sciatic nerve ligation, spared nerve injury, and CCI did not alter results in the OF or EPM in mice [34, 35]. However, other studies assessing behavior at later time points after nerve lesion reported increased anxiety-like behavior also in mice [36, 37]. Also, age-related differences in anxiety-like behavior have previously been shown in 2–12 months old mice [38]. This might explain the lack of intergroup differences in the LDB, OF, and EPM tests in our young mouse cohort. However, we found that B7-H1 ko mice travel longer distances in the center zone of the OF. But, since the intra-individual variability is high in tests for affective behavior, we applied three different paradigms to cover several facets and one single result should not be considered as sufficient to conclude anxiety-like behavior [39].

Peripherally produced cytokines are involved in the development of pain and “sickness behavior” [40], which is characterized by fever, but also by behavioral changes of patients and sick animals such as lethargy, loss of appetite, and depressive symptoms [6]. Several studies also reported that chronic neuroinflammation leads to cognitive deficits in animal models. Intraperitoneal interleukin-1 beta (IL-1β) injection led to spatial learning impairment in mice, while treatment with anti-IL-1β antibodies normalized learning behavior [41]. Belarbi and colleagues reversed chronic neuroinflammation induced cognitive impairment during novel place recognition and spatial learning in rats by treatment with a tumor necrosis factor protein synthesis inhibitor. Interestingly, chronic inflammation had no influence on novel object recognition [42].

In a recent study IL-18 deficient mice showed a schizophrenia-like phenotype (higher impulsivity and aggression), but no relevant behavioral changes in tests for anxiety- (EPM) or depression-like behavior (FST) [43]. Also, tumor necrosis factor ko mice did not show alterations in anxiety- and depression-related behavior in the EPM and FST [44]. This is in line with our results that did not show anxiety, depression, or cognitive deficits in the majority of tests in B7-H1 ko mice despite their pro-inflammatory phenotype. One reason might be that PD-L2 as another PD-1 ligand compensates the lack of B7-H1, due to its overlapping expression pattern and similar function. PD-1 engagement by programmed death ligand 2 (PD-L2), and B7-H1 for example leads to a downregulation of TCR-CD28-stimulation of cytokine production [45].

Our study has several limitations. We did not perform tests making use of aversive behavior like the place preference test or fear conditioning. Tests applied for affective behavior required an external stimulus and measure evoked behavior, which may have masked hints for spontaneous pain. Furthermore, we performed tests for affective and cognitive behavior during the first four weeks after pain induction, which may have been too early [37].

Despite these limitations, our study provides insights into affective and cognitive behavior of B7-H1 ko mice after CCI. We performed three different exploration-based assays [39]; each mouse task differed in environmental conditions, which allows elucidating affective behavior in distinct settings and at different time points. To assess complementary aspects of cognition, we applied two different tests, the Morris water maze for spatial learning and memory and the novel object recognition test for object recognition, long- and short-term memory [22, 46].

## Conclusions

As previously shown for spared nerve injury [12], affective and cognitive behaviour in B7-H1 ko mice was also not influenced by CCI, a model in which recovery from the pain state is different between the genotypes. Our results indicate that the impact of B7-H1 on affective-, depression-like- and learning-behavior, and memory performance might be of minor relevance in mice after nerve lesion.

## Abbreviations

B7-H1: B7 homolog 1
CCI: chronic constriction injury
EPM: elevated plus maze
FST: forced-swim test
IL-1β: interleukin-1 beta
LDB: light–dark box
MWM: morris water maze
NOR: novel object recognition
OF: open field
PD-1: programmed-death receptor-1
PD-L2: programmed death ligand 2
PT: probe trial
SNI: spared nerve injury
WT: wildtype littermates

## References

[1] Treede RD, Jensen TS, Campbell JN, Cruccu G, Dostrovsky JO, Griffin JW (2008). Neuropathic pain: redefinition and a grading system for clinical and research purposes. Neurology..

[2] Attal N, Lanteri-Minet M, Laurent B, Fermanian J, Bouhassira D (2011). The specific disease burden of neuropathic pain: results of a French nationwide survey. Pain.

[3] Duenas M, Ojeda B, Salazar A, Mico JA, Failde I (2016). A review of chronic pain impact on patients, their social environment and the health care system. J Pain Res..

[4] Ellis A, Bennett DL (2013). Neuroinflammation and the generation of neuropathic pain. Br J Anaesth.

[5] Calvo M, Dawes JM, Bennett DL (2012). The role of the immune system in the generation of neuropathic pain. Lancet Neurol.

[6] Dantzer R, O’Connor JC, Freund GG, Johnson RW, Kelley KW (2008). From inflammation to sickness and depression: when the immune system subjugates the brain. Nat Rev Neurosci.

[7] Lyman M, Lloyd DG, Ji X, Vizcaychipi MP, Ma D (2014). Neuroinflammation: the role and consequences. Neurosci Res.

[8] Dong H, Zhu G, Tamada K, Chen L (1999). B7-H1, a third member of the B7 family, co-stimulates T-cell proliferation and interleukin-10 secretion. Nat Med.

[9] Ostrand-Rosenberg S, Horn LA, Haile ST (2014). The programmed death-1 immune-suppressive pathway: barrier to antitumor immunity. J Immunol..

[10] Coyle AJ, Gutierrez-Ramos JC (2001). The expanding B7 superfamily: increasing complexity in costimulatory signals regulating T cell function. Nat Immunol.

[11] Üçeyler N, Göbel K, Meuth SG, Ortler S, Stoll G, Sommer C (2010). Deficiency of the negative immune regulator B7-H1 enhances inflammation and neuropathic pain after chronic constriction injury of mouse sciatic nerve. Exp Neurol.

[12] Karl F, Griesshammer A, Uceyler N, Sommer C (2017). Differential Impact of miR-21 on Pain and Associated Affective and Cognitive Behavior after Spared Nerve Injury in B7-H1 ko Mouse. Front Mol Neurosci..

[13] Chen G, Kim YH, Li H, Luo H, Liu DL, Zhang ZJ (2017). PD-L1 inhibits acute and chronic pain by suppressing nociceptive neuron activity via PD-1. Nat Neurosci.

[14] Dong H, Zhu G, Tamada K, Flies DB, van Deursen JM, Chen L (2004). B7-H1 determines accumulation and deletion of intrahepatic CD8(+) T lymphocytes. Immunity.

[15] Karl F, Griesshammer A, Üçeyler N, Sommer C (2017). Differential Impact of miR-21 on Pain and Associated Affective and Cognitive Behavior after Spared Nerve Injury in B7-H1 ko Mouse. Front Mol Neurosci..

[16] Chaplan SR, Bach FW, Pogrel JW, Chung JM, Yaksh TL (1994). Quantitative assessment of tactile allodynia in the rat paw. J Neurosci Methods.

[17] Hargreaves K, Dubner R, Brown F, Flores C, Joris J (1988). A new and sensitive method for measuring thermal nociception in cutaneous hyperalgesia. Pain.

[18] Crawley J, Goodwin FK (1980). Preliminary report of a simple animal behavior model for the anxiolytic effects of benzodiazepines. Pharmacol Biochem Behav.

[19] Pellow S, Chopin P, File SE, Briley M (1985). Validation of open:closed arm entries in an elevated plus-maze as a measure of anxiety in the rat. J Neurosci Methods.

[20] Prut L, Belzung C (2003). The open field as a paradigm to measure the effects of drugs on anxiety-like behaviors: a review. Eur J Pharmacol.

[21] Porsolt RD, Bertin A, Jalfre M (1977). Behavioral despair in mice: a primary screening test for antidepressants. Arch Int Pharmacodyn Ther.

[22] Morris R (1984). Developments of a water-maze procedure for studying spatial learning in the rat. J Neurosci Methods.

[23] Antunes M, Biala G (2012). The novel object recognition memory: neurobiology, test procedure, and its modifications. Cogn Process.

[24] Vogel-Ciernia A, Wood MA (2014). Examining object location and object recognition memory in mice. Curr Protoc Neurosci.

[25] Botton PH, Costa MS, Ardais AP, Mioranzza S, Souza DO, da Rocha JB (2010). Caffeine prevents disruption of memory consolidation in the inhibitory avoidance and novel object recognition tasks by scopolamine in adult mice. Behav Brain Res.

[26] Bennett GJ, Xie YK (1988). A peripheral mononeuropathy in rat that produces disorders of pain sensation like those seen in man. Pain.

[27] Sommer C, Schäfers M (1998). Painful mononeuropathy in C57BL/Wld mice with delayed wallerian degeneration: differential effects of cytokine production and nerve regeneration on thermal and mechanical hypersensitivity. Brain Res.

[28] Moriarty O, McGuire BE, Finn DP (2011). The effect of pain on cognitive function: a review of clinical and preclinical research. Prog Neurobiol.

[29] Radat F, Margot-Duclot A, Attal N (2013). Psychiatric co-morbidities in patients with chronic peripheral neuropathic pain: a multicentre cohort study. Eur J Pain.

[30] Leite-Almeida H, Almeida-Torres L, Mesquita AR, Pertovaara A, Sousa N, Cerqueira JJ (2009). The impact of age on emotional and cognitive behaviours triggered by experimental neuropathy in rats. Pain.

[31] Yalcin I, Barthas F, Barrot M (2014). Emotional consequences of neuropathic pain: insight from preclinical studies. Neurosci Biobehav Rev.

[32] Roeska K, Ceci A, Treede RD, Doods H (2009). Effect of high trait anxiety on mechanical hypersensitivity in male rats. Neurosci Lett.

[33] Fukuhara K, Ishikawa K, Yasuda S, Kishishita Y, Kim HK, Kakeda T (2012). Intracerebroventricular 4-methylcatechol (4-MC) ameliorates chronic pain associated with depression-like behavior via induction of brain-derived neurotrophic factor (BDNF). Cell Mol Neurobiol.

[34] Hasnie FS, Wallace VC, Hefner K, Holmes A, Rice AS (2007). Mechanical and cold hypersensitivity in nerve-injured C57BL/6J mice is not associated with fear-avoidance- and depression-related behaviour. Br J Anaesth.

[35] Urban R, Scherrer G, Goulding EH, Tecott LH, Basbaum AI (2011). Behavioral indices of ongoing pain are largely unchanged in male mice with tissue or nerve injury-induced mechanical hypersensitivity. Pain.

[36] Palazzo E, Luongo L, Guida F, Marabese I, Romano R, Iannotta M (2016). D-Aspartate drinking solution alleviates pain and cognitive impairment in neuropathic mice. Amino Acids.

[37] Yalcin I, Bohren Y, Waltisperger E, Sage-Ciocca D, Yin JC, Freund-Mercier MJ (2011). A time-dependent history of mood disorders in a murine model of neuropathic pain. Biol Psychiatry.

[38] Shoji H, Takao K, Hattori S, Miyakawa T (2016). Age-related changes in behavior in C57BL/6J mice from young adulthood to middle age. Mol Brain..

[39] Ramos A (2008). Animal models of anxiety: do I need multiple tests?. Trends Pharmacol Sci.

[40] D’Mello C, Ronaghan N, Zaheer R, Dicay M, Le T, MacNaughton WK (2015). Probiotics Improve Inflammation-Associated Sickness Behavior by Altering Communication between the Peripheral Immune System and the Brain. J Neurosci.

[41] Gibertini M, Newton C, Friedman H, Klein TW (1995). Spatial learning impairment in mice infected with Legionella pneumophila or administered exogenous interleukin-1-beta. Brain Behav Immun.

[42] Belarbi K, Jopson T, Tweedie D, Arellano C, Luo W, Greig NH (2012). TNF-alpha protein synthesis inhibitor restores neuronal function and reverses cognitive deficits induced by chronic neuroinflammation. J Neuroinflammation..

[43] Lisboa SF, Issy AC, Biojone C, Montezuma K, Fattori V, Del-Bel EA (2018). Mice lacking interleukin-18 gene display behavioral changes in animal models of psychiatric disorders: possible involvement of immunological mechanisms. J Neuroimmunol.

[44] Fursenko DV, Khotskin NV, Kulikov VA (2016). Behavioral phenotyping of mice deficient in the tumor necrosis factor. Russ J Genet Appl Res.

[45] Latchman Y, Wood CR, Chernova T, Chaudhary D, Borde M, Chernova I (2001). PD-L2 is a second ligand for PD-1 and inhibits T cell activation. Nat Immunol.

[46] Bevins RA, Besheer J (2006). Object recognition in rats and mice: a one-trial non-matching-to-sample learning task to study ‘recognition memory’. Nat Protoc.

